# HCSG-SLAM: Lightweight History-Consistent Semantic Fusion for Gaussian SLAM with Noisy Semantic Predictions

**DOI:** 10.3390/s26206507

**Published:** 2026-10-14

**Authors:** Chen Dai, Haoyu Duanmu, Xu Sun, Dian Deng, Youmei Han

**Affiliations:** 1 School of Marine Technology and Geomatics, Jiangsu Ocean University, Lianyungang 222005, China; 2024220203@jou.edu.cn (C.D.); 2024220204@jou.edu.cn (H.D.); 2025220227@jou.edu.cn (X.S.); 2025210245@jou.edu.cn (D.D.); 2 Advanced Manufacturing College, Beijing Institute of Economics and Management, Beijing 100018, China

**Keywords:** 3D Gaussian Splatting, Gaussian SLAM, semantic fusion, temporal semantic consistency, noisy semantic predictions

## Abstract

Online semantic Gaussian SLAM typically updates three-dimensional semantic maps using frame-wise predictions from two-dimensional semantic segmentation networks. However, occlusions, viewpoint changes, and object boundaries may introduce transient misclassifications, which can further cause cross-frame semantic inconsistency and error propagation. To address this issue, we propose HCSG-SLAM (History-Consistent Semantic Gaussian SLAM), a lightweight history-consistent semantic fusion method for Gaussian SLAM that introduces a compact Gaussian-level historical semantic state without modifying the main RGB/depth tracking and reconstruction pipeline of SGS-SLAM. Each Gaussian maintains only a candidate semantic label, a voting state, and an effective historical observation count. A streaming voting strategy forms the historical semantic consensus, while a strictly causal target-before-current-update strategy prevents the current observation from contributing to its own correction target. Experiments on Replica and ScanNet used segmentation-network predictions rather than ground-truth semantics as input. On Replica, the mean mIoU increased from 0.300830 to 0.307010, with positive gains in all 15 paired runs. On ScanNet, the evaluation was extended to five scenes, where the overall mean mIoU increased from 0.366279 to 0.372166, with positive gains in 22 out of 25 paired scene–seed runs. Per-class analysis shows that the gains were concentrated in several structural and furniture categories, while a small number of classes remained unchanged or slightly decreased. Positive gains were also observed under all nine controlled transient-corruption settings, and additional persistent class-confusion and structured boundary-shift tests yielded positive or comparable performance across the evaluated scenes. Compared with FD-EMA, HCSG-SLAM achieved comparable semantic accuracy while requiring only 12 B of additional temporal state per Gaussian compared with approximately 202 B for FD-EMA. HCSG-SLAM introduced approximately 2.05% additional mapping time relative to the Baseline while leaving tracking essentially unchanged. These results indicate that compact Gaussian-level temporal semantic states can improve semantic consistency and robustness with modest online fusion overhead.

## 1. Introduction

Simultaneous Localization and Mapping (SLAM) jointly estimates the pose of a camera or other onboard sensors while constructing a map of the surrounding environment, thereby providing a fundamental spatial representation for robotic navigation, augmented reality, digital twins, and scene understanding. Conventional geometric SLAM primarily addresses where the sensor is and what the geometry of the environment is, whereas semantic SLAM further aims to describe what exists in the environment by associating spatial primitives with semantic categories, object identities, or higher-level scene information.

In recent years, SLAM systems based on 3D Gaussian representations have gradually extended from conventional indoor environments to more challenging application scenarios. SonarReg-GS SLAM incorporates sparse sonar depth constraints to improve scale and geometric estimation in underwater Gaussian SLAM [1]. DGOMapping employs a 4D Gaussian representation for real-time multi-agent mapping [2]. For dynamic environments, LCD-Splat combines dynamic-object handling, Gaussian submaps, and loop-closure constraints to improve global map consistency [3]. BDGS-SLAM further enhances robustness in dynamic scenes through probabilistic modeling [4]. Chen et al. integrated visual SLAM with 3D Gaussian Splatting (3DGS) reconstruction and introduced a coarse-to-fine dynamic-region segmentation strategy to reduce the influence of moving objects on mapping [5]. SGF-SLAM investigates semantic Gaussian filtering for SLAM in urban road environments [6]. Beyond SLAM, Scaffold-GS introduces anchor-based structured Gaussians together with adaptive anchor growing and pruning to reduce redundant Gaussian primitives while maintaining high-quality rendering [7]. These studies demonstrate the increasing applicability of Gaussian representations to complex robotic mapping scenarios.

3D Gaussian Splatting was originally introduced by Kerbl et al. [8], using explicit anisotropic Gaussian primitives and differentiable splatting to achieve high-quality real-time novel-view rendering. Building on this representation, GS-SLAM introduces 3D Gaussians into dense visual SLAM and develops corresponding Gaussian expansion and camera-pose optimization strategies [9]. Gaussian Splatting SLAM further demonstrates the feasibility of using Gaussian primitives as a unified explicit scene representation for online tracking, mapping, and rendering [10]. SplaTAM develops an online tracking-and-mapping framework based on 3D Gaussians for RGB-D input [11], while Photo-SLAM supports monocular, stereo, and RGB-D cameras for real-time localization and photorealistic mapping [12].

Semantic information has subsequently been incorporated into Gaussian-based SLAM. SGS-SLAM integrates semantic features into multi-channel Gaussian optimization and uses semantic information to support dense semantic mapping [13]. SemGauss-SLAM embeds semantic features into Gaussian representations and further exploits semantic information during bundle adjustment [14]. ReSemGS-SLAM aggregates multi-frame observations to estimate per-Gaussian class distributions and introduces semantic-consistency mechanisms to improve cross-frame stability [15]. GSFF-SLAM adopts a high-dimensional feature field and specifically addresses semantic reconstruction under sparse or noisy two-dimensional semantic priors [16]. Despite these advances, the trade-off between the temporal stabilization of imperfect frame-wise semantic predictions and per-Gaussian semantic-state cost remains insufficiently explored. Existing approaches often rely on complete class distributions or high-dimensional feature representations, which introduce additional state and computational overhead. This motivates the development of a compact historical representation that can exploit temporal semantic evidence without maintaining a complete class distribution.

To address this problem, we propose HCSG-SLAM (History-Consistent Semantic Gaussian SLAM), a lightweight history-consistent semantic fusion approach that augments SGS-SLAM with a compact Gaussian-level historical semantic state and a strictly causal semantic correction mechanism while preserving the main RGB/depth tracking and reconstruction pipeline of SGS-SLAM. The main contributions of this work are summarized as follows:

(1) We introduce a lightweight Gaussian-level historical semantic state that aggregates multi-frame semantic observations using only a small number of scalar variables, avoiding the need to maintain a complete semantic distribution for each Gaussian. The component-level ablation further identifies persistent candidate-and-vote accumulation as the principal source of the observed semantic accuracy improvement.
(2) We propose a strictly causal history-consistent semantic correction strategy, in which the current semantic prediction is corrected only using historical information accumulated from previous frames, thereby reducing transient semantic errors and avoiding self-reinforcement from the current observation.
(3) We conduct extensive evaluations on Replica and five ScanNet scenes using segmentation-network predictions rather than perfect semantic labels. Multi-seed experiments, scene-level and per-class analyses, comparisons with multiple temporal-fusion references, controlled transient corruption, persistent class confusion, structured boundary perturbations, and efficiency profiling are used to characterize the semantic accuracy, robustness, and state-efficiency trade-offs of HCSG-SLAM.

## 2. Related Work

### 2.1. 3D Gaussian Splatting and Gaussian SLAM

3D Gaussian Splatting (3DGS) provides an explicit, differentiable, and rendering-efficient scene representation that is well-suited to online dense mapping. GS-SLAM extends 3DGS to dense visual SLAM by jointly optimizing Gaussian map construction and camera tracking. Gaussian Splatting SLAM further demonstrates the feasibility of using Gaussian primitives as a unified representation for online tracking, mapping, and rendering. SplaTAM develops an explicit 3D Gaussian tracking-and-mapping framework for RGB-D SLAM, whereas Photo-SLAM combines geometric features with photorealistic Gaussian mapping and supports monocular, stereo, and RGB-D inputs.

Beyond local tracking and mapping, recent studies have also addressed global consistency and learned tracking. Splat-SLAM introduces global optimization for RGB-only Gaussian SLAM [17], while DROID-Splat combines an end-to-end visual tracker with a 3DGS renderer to integrate learned tracking with Gaussian-based mapping [18]. A recent survey further summarizes the development of 3DGS in robotics, 3D reconstruction, and navigation [19]. Collectively, these studies establish 3D Gaussians as an effective scene representation for online SLAM, but their primary focus is geometric reconstruction, camera tracking, and global consistency rather than the temporal stabilization of noisy semantic predictions.

### 2.2. Semantic Gaussian SLAM

Semantic information has increasingly been incorporated into Gaussian-based SLAM. SGS-SLAM jointly optimizes appearance, geometry, and semantic features within a multi-channel Gaussian representation and introduces semantic losses and keyframe mechanisms for dense semantic mapping. SemGauss-SLAM embeds semantic features into Gaussian primitives and further uses semantic information to assist bundle adjustment. ReSemGS-SLAM aggregates weighted multi-frame observations to construct per-Gaussian class distributions and introduces semantic-consistency constraints to improve cross-frame stability. GSFF-SLAM represents semantic information using a high-dimensional feature field and specifically addresses sparse or noisy 2D semantic priors.

Beyond online SLAM, the storage and computational cost of high-dimensional semantic Gaussian representations has also attracted increasing attention. DF-3DGS reduces the cost of semantic features through feature decoupling and hierarchical compression [20]. Feature 3DGS attaches distilled high-dimensional visual features to Gaussian primitives to construct renderable feature fields [21], while LangSplat further embeds language features into 3D Gaussians for open-vocabulary 3D scene understanding [22].

These methods demonstrate that Gaussian primitives can effectively carry semantic or high-dimensional features. However, many existing approaches rely on complete class distributions or high-dimensional feature representations, whereas HCSG-SLAM focuses on whether cross-frame semantic consistency can be improved using only a compact, fixed-size historical state.

### 2.3. Comparison with Existing Multi-Frame Semantic Fusion Methods

Multi-frame semantic fusion is not unique to Gaussian SLAM. Conventional semantic SLAM systems have developed various strategies for integrating frame-wise 2D semantic observations into persistent 3D maps. DS-SLAM combines semantic segmentation with motion-consistency checking to suppress dynamic features while constructing dense semantic maps in dynamic environments [23]. DynaSLAM combines dynamic-object detection with geometric reasoning to improve camera tracking and static-scene mapping in dynamic environments [24]. MaskFusion uses instance-level semantic segmentation to recognize, track, and reconstruct multiple independently moving objects while maintaining an object-aware semantic map [25]. PanopticFusion integrates frame-wise semantic and instance predictions into a persistent volumetric map while maintaining cross-frame consistency of semantic and instance information [26]. Semantic Fusion represents a representative probabilistic fusion approach in which CNN-based semantic predictions from multiple views are continuously integrated into a persistent 3D map through dense geometric correspondences [27].

More broadly, tightly coupled LiDAR–visual–inertial systems such as LVI-SAM demonstrate how complementary sensing modalities can be integrated to improve robust state estimation and mapping [28]. ORB-SLAM3 demonstrates robust tracking, relocalization, and map reuse in visual, visual–inertial, and multi-map settings [29], whereas NICE-SLAM investigates scalable dense scene representation and online tracking and mapping using neural implicit representations [30]. These studies address different aspects of SLAM system design and should not be regarded as semantic fusion methods themselves.

Li et al. [31] investigated SLAM robustness from the sensor-degradation perspective and proposed an adaptive compensation strategy that estimates the degree of sensor degradation and dynamically adjusts the contribution of different sensor measurements during pose optimization. This approach addresses robustness at the sensor and pose-estimation level, whereas HCSG-SLAM focuses on a different source of degradation, namely temporally inconsistent semantic predictions propagated into the Gaussian semantic map. The two approaches are therefore complementary: degradation-aware sensor compensation improves the reliability of geometric localization inputs, while HCSG-SLAM improves the temporal consistency of semantic supervision without modifying the original RGB/depth tracking pipeline.

In contrast to the above approaches, HCSG-SLAM addresses a more specific problem: when frame-wise predictions from a 2D semantic segmentation network are used as online supervision in Gaussian SLAM, can temporal semantic evidence be retained using only a fixed number of Gaussian-level scalar states while correcting transient conflicting observations without maintaining a complete class distribution? HCSG-SLAM addresses this question through a compact historical semantic state and a strictly causal correction mechanism, in which only information accumulated from previous frames is used to modify the current semantic supervision.

Direct numerical comparison with published semantic Gaussian SLAM systems is not straightforward because these methods employ different semantic representations, supervision sources, and optimization pipelines. For example, ReSemGS-SLAM maintains per-Gaussian semantic distributions, whereas GSFF-SLAM adopts a high-dimensional feature-field representation. Their reported results are therefore not directly mixed with our controlled quantitative comparison. Instead, to isolate the effect of temporal semantic fusion under identical semantic inputs and SLAM settings, we additionally compare HCSG-SLAM with several controlled temporal-fusion references, including histogram-based majority fusion, sliding-window majority fusion, confidence-weighted fusion, and full-distribution exponential moving average fusion. All of these references use the same cached semantic predictions, scenes, frame ranges, and SGS-SLAM backbone as HCSG-SLAM.

## 3. Methodology

### 3.1. System Overview and Problem Formulation

Let the online RGB-D sequence be denoted as:

(1) $${\left\{I_{t},D_{t}\right\}}_{t=1}^{T}$$

where *I_t_* and *D_t_* denote the RGB image and depth map at frame *t*, respectively, and *T* is the total number of frames. Given *I_t_*, a 2D semantic segmentation network produces the pixel-wise discrete semantic prediction $y_{t}^{\mathrm{obs}}(x)$, where *x* denotes the pixel location. To remain consistent with the semantic mapping branch of SGS-SLAM, a fixed semantic encoding function *Φ* is used to map the discrete semantic label to its semantic supervision representation:

(2) $$S_{t}^{\mathrm{obs}}(x)=\Phi (y_{t}^{\mathrm{obs}}(x))$$

Here, $S_{t}^{\mathrm{obs}}(x)$ denotes the encoded semantic supervision at pixel *x*. HCSG-SLAM retains this semantic representation and the original RGB/Depth tracking and Gaussian mapping pipeline of SGS-SLAM, and introduces the proposed history-consistent semantic fusion mechanism only into the semantic mapping branch.

**Figure 1:**
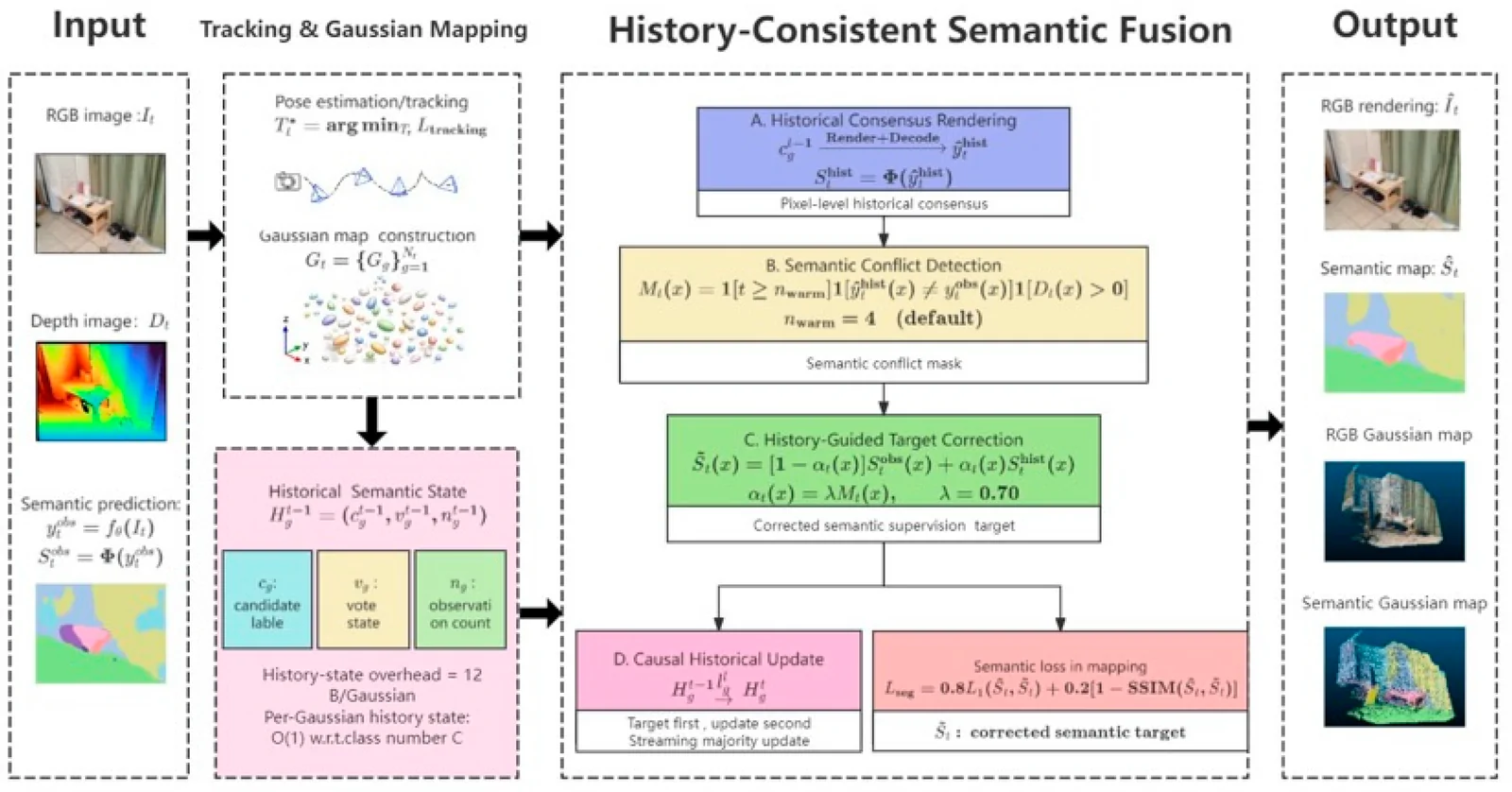
Overall framework of the proposed HCSG-SLAM.

The overall framework of HCSG-SLAM is shown in Figure 1. Given the RGB image, depth map, and network semantic prediction, the original SGS-SLAM pipeline performs camera tracking and online Gaussian map construction. In parallel, HCSG-SLAM maintains a compact historical semantic state for each Gaussian. These historical states are rendered into the current camera view to construct a pixel-level historical consensus, which is then used to detect semantic conflicts and generate a history-guided correction target. Importantly, the current semantic observation is written into the historical state only after the correction target has been constructed, thereby preserving the strictly causal target-before-current-update order. The corrected semantic target is subsequently used only in the semantic mapping loss, while the original RGB, depth, and tracking optimization terms remain unchanged.

### 3.2. Lightweight Gaussian-Level Historical Semantic State

For each Gaussian *g*, HCSG-SLAM maintains a compact historical semantic state at time *t*, defined as:

(3) $$H_{g}^{t}=\left(c_{g}^{t},v_{g}^{t},n_{g}^{t}\right)$$

where $c_{g}^{t}$ denotes the candidate semantic label, $v_{g}^{t}$ denotes the candidate voting state, and $n_{g}^{t}$ denotes the number of valid historical semantic observations associated with Gaussian *g*. In the current implementation, all three variables are stored in float32 format, resulting in an additional historical-state cost of 3 × 4 = 12 bytes per Gaussian.

The historical state has a fixed dimensionality with respect to the number of semantic classes *C*. In other words, the number of additional scalar variables stored for each Gaussian does not increase as *C* grows. Therefore, the *O*(1) notation used in this work refers specifically to the per-Gaussian historical-state dimensionality with respect to *C*, rather than to the total memory consumption of the complete Gaussian map. The online update rule for this historical state is described in Section 3.3.

### 3.3. Streaming Historical Candidate Update

Before applying the streaming historical update, HCSG-SLAM establishes a depth-consistent association between the current 2D semantic prediction and each Gaussian primitive. Specifically, the center of Gaussian *g* is first transformed into the current camera coordinate system and projected onto the image plane, yielding the projected pixel $\left(u_{g}^{t,\mathrm{proj}},v_{g}^{t,\mathrm{proj}}\right)$ and camera-space depth $z_{g}^{t}$. A Gaussian is considered a valid candidate only when it lies in front of the camera and its projected pixel falls inside the image. The measured depth $D_{t}\left(u_{g}^{t,\mathrm{proj}},v_{g}^{t,\mathrm{proj}}\right)$ is then sampled at the projected location, and the semantic observation is accepted only if the measured depth is valid and satisfies:

(4) $$\left|z_{g}^{t}-D_{t}\left(u_{g}^{t,\mathrm{proj}},v_{g}^{t,\mathrm{proj}}\right)\right|\leq \delta_{\mathrm{abs}}+\delta_{\mathrm{rel}}\left|D_{t}\left(u_{g}^{t,\mathrm{proj}},v_{g}^{t,\mathrm{proj}}\right)\right|$$

In our implementation, depth values are expressed in meters, with the absolute depth tolerance set to *δ_abs_* = 0.05*m* and the dimensionless relative tolerance set to *δ_rel_* = 0.05. The absolute tolerance accounts for small depth discrepancies independently of the observed distance, whereas the relative tolerance allows the admissible discrepancy to increase proportionally with depth. Their combination provides a depth-dependent acceptance criterion for associating projected Gaussian centers with observed surfaces. These values are fixed across all experiments without scene-specific adjustment. The depth-consistency test suppresses associations with Gaussians that are geometrically inconsistent with the currently observed surface, thereby reducing erroneous semantic updates caused by occlusion.

To reduce ambiguous associations near semantic boundaries, pixels located on class discontinuities are excluded from the historical-state update. The boundary mask is obtained from four-neighbor label changes and is expanded using a 3 × 3 max-pooling operation. The semantic label at the projected pixel is then assigned to the corresponding Gaussian only when both the depth-consistency and non-boundary conditions are satisfied. This 2D-to-Gaussian association does not select a single Gaussian according to opacity or alpha-contribution weight; instead, each projected Gaussian is independently tested against the observed depth. Consequently, multiple Gaussians may project to the same image pixel, but only those consistent with the observed surface are retained. Conversely, because the center of each Gaussian projects to a single pixel, each Gaussian receives at most one discrete semantic observation from the current frame, and no additional within-frame aggregation is required. Temporal aggregation is subsequently performed across frames by the streaming candidate-and-vote update described below.

It should be distinguished from the reverse historical-consensus rendering described in Section 3.4, where Gaussian-level historical states are projected back into the image using the standard Gaussian rasterization and compositing process.

For each valid semantic observation associated with Gaussian *g* at frame *t*, let the observed discrete semantic class be denoted by the symbol already defined in the preceding formulation. To avoid storing a *C*-dimensional class-probability vector for every Gaussian, HCSG-SLAM adopts a Boyer–Moore-style streaming candidate update [32]. The temporary voting state is first updated according to whether the current semantic observation agrees with the historical candidate:

(5) $${\bar{v}}_{g}^{t}=\left\{\begin{array}{c} v_{g}^{t-1}+1,\;l_{g}^{t}=c_{g}^{t-1}\\ v_{g}^{t-1}-1,l_{g}^{t}\neq c_{g}^{t-1} \end{array}\right.$$

If the updated vote becomes non-positive, the current semantic observation is used to establish a new candidate; otherwise, the previous historical candidate is retained:

(6) $$(c_{g}^{t},\;v_{g}^{t})=\left\{\begin{array}{c} (l_{g}^{t},1),{\bar{v}}_{g}^{t}\leq 0\\ (c_{g}^{t-1},{\bar{v}}_{g}^{t}),{\bar{v}}_{g}^{t}>0 \end{array}\right.$$

Specifically, when the current semantic observation agrees with the historical candidate, the temporary voting state is increased by one, thereby strengthening the accumulated support for the current candidate. When the observation conflicts with the candidate, the voting state is decreased by one. Candidate replacement occurs only when the accumulated voting margin is exhausted, i.e., when the temporary vote becomes non-positive. In this case, the current observation is adopted as the new candidate and the voting state is reset to one. Therefore, a single conflicting observation cannot immediately overturn a candidate that has accumulated sufficient historical support.

For each valid Gaussian–semantic association, the number of historical semantic observations is incremented accordingly:

(7) $$n_{g}^{t}=n_{g}^{t-1}+1$$

As a simple example, suppose the historical candidate is class *A* with an initial voting state of 1, and the subsequent semantic observations are *A*, *A*, *B*, *A*. According to Equations (5) and (6), the voting state evolves as 2 → 3 → 2 → 3, while the candidate remains *A* throughout the sequence. Thus, the single conflicting observation *B* only reduces the accumulated support from 3 to 2 and does not immediately replace the historically supported candidate.

This streaming update maintains only a dominant semantic candidate and its relative support state, without storing a complete class histogram or a *C*-dimensional probability distribution. It therefore provides a compact mechanism for maintaining semantic history in an online Gaussian map. It should be emphasized that the Boyer–Moore-style update is used here only to track a dominant candidate in a streaming manner; it does not guarantee recovery of the true majority class for an arbitrary sequence of semantic observations.

### 3.4. Historical Consensus Rendering

After the Gaussian-level historical candidate states have been accumulated up to frame *t* − 1, these discrete candidates need to be projected into the current camera view at frame *t* to construct a pixel-level historical semantic consensus. HCSG-SLAM performs historical candidate rendering and class decoding using the current camera pose *T_t_*, the camera intrinsic matrix *K*, and the historical candidate labels $c_{g}^{t-1}$ of the Gaussian primitives:

(8) $${\hat{y}}_{t}^{\mathrm{hist}}(x)=R_{\mathrm{dec}}({\left\{c_{g}^{t-1}\right\}}_{g=1}^{N};T_{t},K,x)$$

where *R_dec_* denotes the render-and-decode operation that projects the historical Gaussian candidates into the current image according to Gaussian visibility and projection relationships, and ${\hat{y}}_{t}^{\mathrm{hist}}(x)$ denotes the resulting historical consensus class at pixel *x*. The same fixed semantic encoding function *Φ* used for the current semantic observation is then applied to convert the historical consensus class into the corresponding semantic supervision representation:

(9) $$S_{t}^{\mathrm{hist}}(x)=\Phi ({\hat{y}}_{t}^{\mathrm{hist}}(x))$$

Importantly, the construction of $S_{t}^{\mathrm{hist}}(x)$ only reads the Gaussian historical states formed at frame *t* − 1 and earlier. The current semantic observation at frame *t* is not used during this process. This temporal separation provides the historical semantic target required for the strictly causal correction procedure introduced in Section 3.5.

### 3.5. Strictly Causal Historical Target Generation

One of the key designs of HCSG-SLAM is to preserve strict causality during the construction of the historical correction target. At frame *t*, the system first reads only the Gaussian historical states that have already been formed up to frame *t* − 1, and uses this previously accumulated history together with the current semantic observation to construct the history-guided corrected target ${\tilde{S}}_{t}$. Only after this target has been constructed is the current semantic observation $l_{g}^{t}$, associated with Gaussian *g*, written into the historical state.

**Figure 2:**
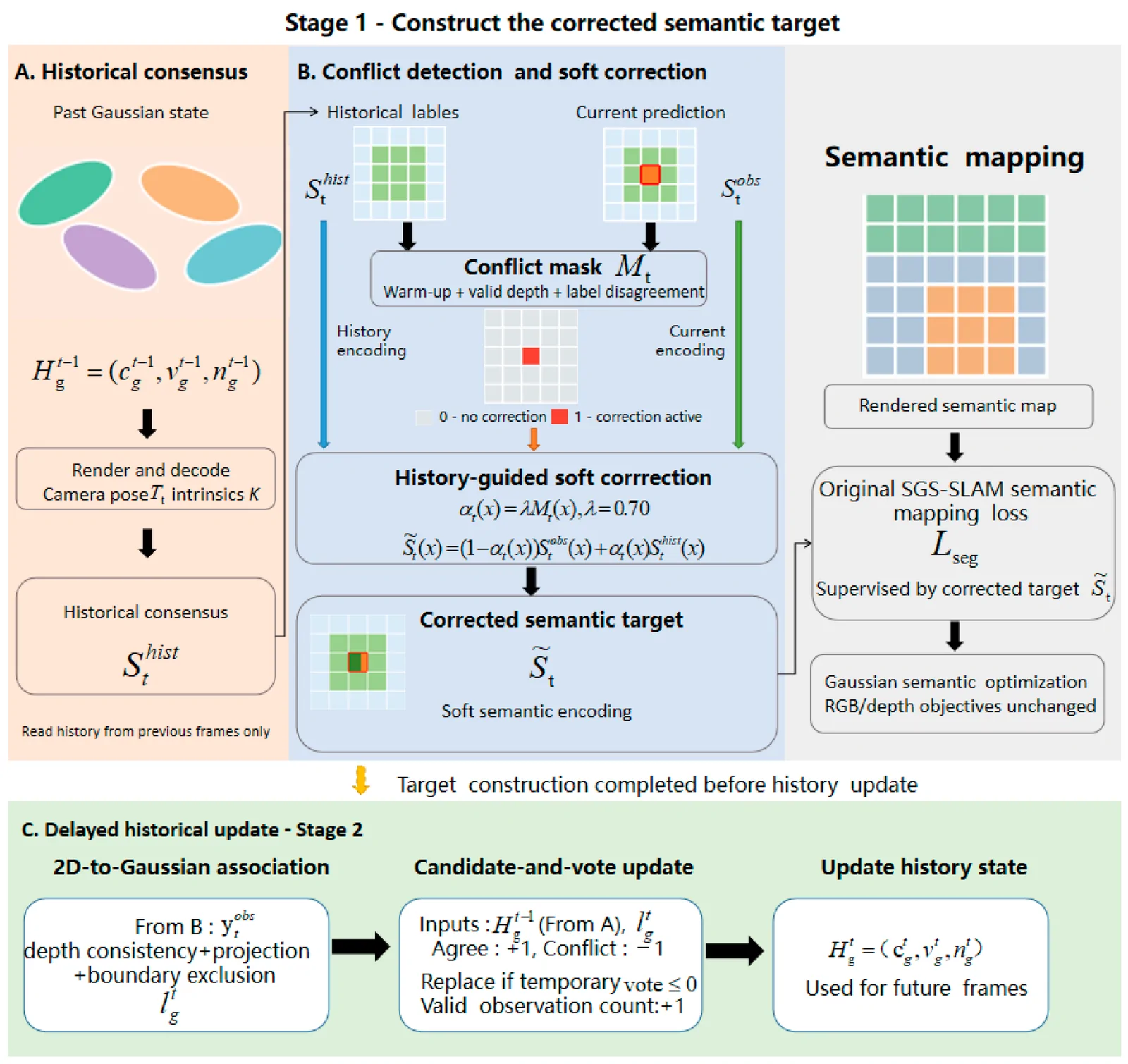
Historical consensus construction and history-guided semantic correction in HCSG-SLAM. The orange, blue, and green backgrounds indicate historical consensus construction, conflict detection and soft correction, and delayed historical update, respectively.

The temporal relationship between historical consensus construction and history-guided semantic correction is illustrated in Figure 2.

To explicitly clarify the strict causal ordering, the corrected semantic target is first constructed using the historical Gaussian states accumulated up to frame *t* − 1 together with the current-frame semantic observations. This process can be summarized as follows:

(10) $$(H^{t-1},S_{t}^{\mathrm{obs}})\overset{\mathrm{Target}\;\mathrm{Construction}}{\to }{\tilde{S}}_{t}$$

Here, $H^{t-1}={\left\{H_{g}^{t-1}\right\}}_{g=1}^{N}$ denotes the collection of Gaussian-level historical semantic states before the current-frame update, where *N* is the number of Gaussians in the current map. In contrast, $H_{g}^{t-1}$ denotes the historical semantic state of an individual Gaussian *g*. The target construction also involves historical-consensus rendering, current depth information, and the camera parameters, as detailed in Section 3.4 and Section 3.6. Only after the corrected target has been constructed are the current valid semantic observations incorporated into the historical states:

(11) $${(H}_{g}^{t-1},l_{g}^{t})\overset{\mathrm{Historical}\;\mathrm{Update}}{\to }H_{g}^{t}$$

The historical state update follows the streaming candidate-and-vote mechanism defined in Equations (5)–(7). This ordering ensures that the current-frame observation is not incorporated into the historical state before its own corrected target is constructed, thereby preventing self-reinforcement through the historical-state update.

### 3.6. Semantic Conflict Detection and History-Guided Soft Correction

The final history-guided correction gate does not use confidence, maturity, or semantic-boundary statistics as additional hard gating criteria. As illustrated in Figure 1 and Figure 2, the history-guided correction at pixel *x* is activated only when three conditions are simultaneously satisfied: (1) the frame-level warm-up has been completed; (2) the current network-predicted class differs from the historical consensus class; and (3) a valid depth observation is available. Accordingly, the binary semantic conflict mask is defined as:

(12) $$M_{t}\left(x\right)=1\left[t\geq n_{\mathrm{warm}}\right]1\left[y_{t}^{\mathrm{obs}}\left(x\right)\neq {\hat{y}}_{t}^{\mathrm{hist}}\left(x\right)\right]1\left[D_{t}\left(x\right)>0\right]$$

Here, 1[∙] denotes the indicator function, *n*_warm_ is the number of warm-up frames, and *D_t_*(*x*) indicates a valid depth observation at pixel *x*. The correction weight is then determined by the conflict mask as:

*α_t_*(*x*) = *λM_t_*(*x*), *λ* = 0.70 (13)

and the history-guided semantic supervision target is constructed as:

(14) $${\tilde{S}}_{t}\left(x\right)=\left[1-\alpha_{t}\left(x\right)\right]S_{t}^{\mathrm{obs}}\left(x\right)+\alpha_{t}\left(x\right)S_{t}^{\mathrm{hist}}(x)$$

When *M_t_*(*x*) = 0, *α_t_*(*x*) = 0, and therefore ${\tilde{S}}_{t}\left(x\right)=S_{t}^{\mathrm{obs}}\left(x\right)$; the semantic supervision remains identical to the current network prediction. When *M_t_*(*x*) = 1, *α_t_*(*x*) = *λ* = 0.70, and the historical semantic target $S_{t}^{\mathrm{hist}}(x)$ contributes to the corrected supervision with a fixed weight. Thus, historical information is introduced only at pixels that satisfy the semantic-conflict conditions, rather than being fused indiscriminately across the entire image.

In all of the main experiments, the fusion coefficient and warm-up length were fixed to *λ* = 0.70 and *n_warm_* = 4, respectively. The same parameter values were used for all Replica and ScanNet scenes and for all random seeds, without dataset-specific or scene-specific tuning. The choice of *λ* = 0.70 provides a history-dominant but still soft correction, rather than completely replacing the current semantic supervision, while *n_warm_* = 4 provides a short initialization period for accumulating historical evidence before history-guided correction is enabled.

Let ${\hat{S}}_{t}$ denote the semantic map rendered from the current Gaussian map at frame *t*. The corrected target ${\tilde{S}}_{t}\left(x\right)$ is subsequently used in the original SGS-SLAM semantic mapping loss:

(15) $$L_{\mathrm{seg}}=0.8L_{1}({\hat{S}}_{t},{\tilde{S}}_{t})+0.2[1-\mathrm{SSIM}({\hat{S}}_{t},{\tilde{S}}_{t})]$$

HCSG-SLAM modifies only the supervision target of the semantic mapping loss and leaves the original RGB, depth, and tracking optimization terms unchanged. Therefore, the proposed mechanism primarily acts on semantic mapping rather than redesigning the frontend pose estimation or geometric reconstruction components.

### 3.7. Gaussian Lifecycle Synchronization and State Complexity

Online Gaussian SLAM continuously creates, prunes, and reorders Gaussian primitives during mapping. To maintain a one-to-one correspondence between the historical semantic states and the underlying Gaussian primitives, the historical state variables *c_g_*, *v_g_*, and *n_g_* are initialized together with each newly created Gaussian. When Gaussian primitives are pruned or their indices are reordered, the same indexing operations are applied to all three historical state variables. Consequently, the proposed historical semantic state remains synchronized with the lifecycle of the online Gaussian map rather than being introduced as an offline post-processing step.

We next analyzed the additional semantic-state memory introduced by HCSG-SLAM. For a map containing *N* Gaussian primitives, each Gaussian stores three float32 historical variables, resulting in:

*M_HCSG_* = 3 × 4*N* = 12*N* bytes (16)

where *M_HCSG_* denotes the additional historical semantic-state memory of HCSG-SLAM. As an internal strong reference, FD-EMA stores a *C*-class float16 distribution for each Gaussian, requiring approximately *2CN* bytes for the class-state representation. The corresponding memory ratio is therefore:

(17) $$\frac{M_{\mathrm{FD}}}{M_{\mathrm{HCSG}}}=\frac{2\mathrm{CN}}{12N}=\frac{C}{6}\approx 16.83$$

Thus, for *C* = 101, FD-EMA requires approximately 16.83 times as much temporal semantic-state memory as HCSG-SLAM under the current representation. Equivalently, HCSG-SLAM requires only approximately 5.94% of the temporal semantic-state memory used by FD-EMA. It should be emphasized that this comparison concerns only the additional Gaussian-level temporal semantic representation and does not represent the total GPU-memory consumption of the complete SLAM process. Accordingly, the term lightweight in this work specifically refers to the compact, fixed-size temporal semantic state and its modest fusion overhead, rather than to minimum whole-process GPU-memory consumption.

## 4. Experiments

### 4.1. Experimental Setup

All experiments were conducted using the official SGS-SLAM codebase (base revision: Git commit e4183986204242a8bb422624618af07780a49d26; https://github.com/ShuhongLL/SGS-SLAM, accessed on 11 October 2026), with the proposed HCSG-SLAM components implemented as local extensions. The hardware platform consisted of an AMD Ryzen 9 7945HX CPU (Advanced Micro Devices, Inc., Santa Clara, CA, USA) with 16 cores and 32 threads and an NVIDIA GeForce RTX 4060 Laptop GPU (NVIDIA Corporation, Santa Clara, CA, USA) with 8188 MiB of memory. The software environment was Ubuntu 22.04.5, Python 3.9.25, and PyTorch 2.0.1 with CUDA 11.8. To ensure a fair comparison, the Baseline and HCSG-SLAM used exactly the same scenes, frame ranges, cached semantic predictions, random seeds, and tracking/mapping parameters. The only difference between the two systems was the proposed historical semantic module. This paired setting was used throughout the main comparison and robustness experiments.

Unless otherwise stated, the five-seed experiments used the fixed random seeds 101, 202, 303, 404, and 505. The controlled temporal-fusion, sensitivity, ablation, and robustness analyses used the explicitly specified scene subset, the first 100 frames, and seed 101. Semantic-network predictions were generated offline and cached before SLAM evaluation so that all paired methods received exactly the same semantic observations.

Experiments were conducted on two publicly available indoor RGB-D datasets, Replica [33] and ScanNet [34]. Replica is a photorealistic synthetic indoor dataset. In our custom split, office1, office2, office3, and room2 were used for semantic-network training, office4 was used for validation, and room0, office0, and room1 were reserved for subsequent SLAM evaluation. The 2D semantic inputs for Replica were generated by DeepLabV3 [35] with a ResNet-50 backbone [36], rather than by using ground-truth semantic labels. ScanNet is a real-world indoor RGB-D dataset. To increase scene-level diversity in the evaluation, the original three-scene protocol was extended to five independent ScanNet scenes: scene0000_00, scene0059_00, scene0106_00, scene0181_00, and scene0207_00. The same DeepLabV3-ResNet50 architecture was used for ScanNet. For ScanNet, the semantic segmentation network was trained on 1197 scenes and validated on 311 separate scenes. The five scenes used for SLAM evaluation, namely scene0000_00, scene0059_00, scene0106_00, scene0181_00, and scene0207_00, were excluded from both the semantic-network training and validation sets. We verified that the training, validation, and evaluation scene sets were mutually disjoint, thereby preventing scene-level overlap between semantic-network development and SLAM evaluation. Network predictions were generated offline and stored in a fixed prediction cache, which was shared by every paired Baseline/HCSG-SLAM run. This design ensures that differences between the two methods are not caused by stochastic variation in semantic-network inference.

For the expanded ScanNet evaluation, repeated random seeds are not treated as independent scene-level samples. Five paired runs are conducted for each scene for descriptive evaluation. For scene-level statistical analysis, the five paired runs within each ScanNet scene are first averaged, yielding one Baseline–HCSG-SLAM difference per scene. Statistical inference is then performed over the resulting five scene-level differences using a paired *t*-test, a Wilcoxon signed-rank test, a 95% confidence interval, and Cohen’s *d_z_*.

Since semantic predictions are precomputed and cached in the main SLAM experiments, the tracking and mapping runtimes reported in the efficiency analysis isolate the online SLAM and temporal-fusion stages and do not directly include semantic-network inference. To additionally characterize semantic-inference-inclusive latency, DeepLabV3-ResNet50 inference was benchmarked separately on the same RTX 4060 Laptop GPU using the first 100 frames of each of the three Replica evaluation scenes, yielding 300 frames in total. The network input resolution was 512 × 288, 20 warm-up iterations were performed before timing, and CUDA synchronization was applied before and after each measured inference. Disk writing of cached predictions was excluded.

### 4.2. Evaluation Metrics and Comparison Methods

Semantic mapping performance is primarily evaluated using the mean Intersection over Union (mIoU). For each valid semantic class *k*, the Intersection over Union (IoU) is computed from the true-positive *TP_k_*, false-positive *FP_k_*, and false-negative *FN_k_* predictions as:

(18) $${\mathrm{IoU}}_{k}=\frac{TP_{k}}{TP_{k}+FP_{k}+FN_{k}}$$

and the mIoU over *K* valid semantic classes is defined as:

(19) $$\mathrm{mIoU}=\frac{1}{K}\sum_{k=1}^{K}{\mathrm{IoU}}_{k}$$

where *K* denotes the number of valid semantic classes included in the evaluation.

In addition to mIoU, a pixel-level transition analysis is used to characterize the direction of semantic changes introduced by HCSG-SLAM. Wrong-to-Right *W* → *R* denotes the proportion of pixels that are incorrectly predicted by the Baseline but correctly predicted by HCSG-SLAM, whereas Right-to-Wrong *R* → *W* denotes the proportion of pixels that are correctly predicted by the Baseline but become incorrect after applying HCSG-SLAM. These two quantities distinguish beneficial error correction from newly introduced semantic errors.

To evaluate whether the proposed semantic correction affects the original SLAM tracking and reconstruction performance, Absolute Trajectory Error (ATE) is used as an auxiliary tracking metric, while Peak Signal-to-Noise Ratio (PSNR), Multi-Scale Structural Similarity Index (MS-SSIM), and Learned Perceptual Image Patch Similarity (LPIPS) are used as auxiliary image-reconstruction metrics. These metrics are not treated as the primary measures of semantic performance; rather, they are used to assess whether the introduction of historical semantic correction causes noticeable degradation in tracking accuracy or photometric and perceptual reconstruction quality.

Six configurations are considered in the comparative evaluation:

Baseline. The frame-wise semantic predictions produced by the segmentation network are directly used as semantic supervision in the original SGS-SLAM mapping pipeline, without any Gaussian-level historical semantic correction.

HCSG-SLAM. This is the proposed method. Each Gaussian maintains three scalar historical state variables, namely the candidate label, voting state, and valid observation count. Semantic correction is performed using the strictly causal historical-target construction strategy described in Section 3, together with semantic-conflict detection and valid-depth checking. Unless otherwise specified in the sensitivity analysis, the fusion coefficient and warm-up length were fixed to *λ* = 0.70 and *n_warm_* = 4, respectively, for all datasets, scenes, and random seeds.

Histogram Majority. For each Gaussian, a cumulative class histogram is maintained over valid semantic observations, and the class with the largest accumulated count is used as the historical semantic consensus. This reference represents an exact cumulative majority-fusion strategy, in contrast to the compact Boyer–Moore-style candidate state used by HCSG-SLAM.

Sliding-Window Majority. Each Gaussian stores the most recent five valid semantic observations, and the majority class within this fixed-length window is used as the historical consensus. This reference evaluates whether a short-term local voting mechanism is sufficient to provide temporal semantic stabilization.

Confidence-Weighted Fusion. Historical class support is accumulated using the confidence associated with each frame-wise semantic prediction rather than treating all valid observations equally. The class with the highest accumulated confidence-weighted support is used as the historical semantic consensus.

FD-EMA (Full-Distribution Exponential Moving Average). FD-EMA is an internal full-distribution reference designed to evaluate the accuracy–state-memory trade-off. For each Gaussian, FD-EMA maintains a complete 101-class semantic probability vector in float16 format and updates this distribution using an exponential moving average with a decay factor of 0.90. In contrast to the compact three-scalar state used by HCSG-SLAM, FD-EMA explicitly preserves the full class distribution for every Gaussian.

The Baseline–HCSG-SLAM comparison evaluates the overall effectiveness of the proposed historical correction mechanism. Histogram Majority, Sliding-Window Majority, and Confidence-Weighted Fusion provide controlled comparisons with alternative temporal aggregation strategies, whereas FD-EMA evaluates whether maintaining a complete semantic class distribution provides a measurable accuracy advantage relative to the compact historical state used by HCSG-SLAM.

### 4.3. Main Quantitative Results and SLAM Preservation

Table 1 summarizes the five-seed quantitative results on Replica and ScanNet. The results are reported using the same fixed semantic prediction cache and paired experimental settings described in Section 4.1. For each scene, the results are reported as mean ± sample standard deviation over five random seeds (*ddof* = 1).

On Replica, HCSG-SLAM improves mIoU in all 15 paired runs across the three test scenes and five random seeds. The overall mean mIoU increases from 0.300830 to 0.307010, corresponding to an absolute improvement of 0.006180 and a relative gain of 2.054%. Across the 15 fixed Baseline–HCSG-SLAM pairs, the mean improvement is 0.006180, with a 95% confidence interval of [0.004451, 0.007909]. Under this paired protocol, a paired *t*-test yields *t*(14) = 7.668, *p* = 2.23 × 10^−6^, with Cohen’s *d_z_* = 1.980. Because these 15 pairs are obtained from repeated runs over three scenes and five random seeds, these statistics are used to characterize the consistency of the observed improvement under the fixed evaluation protocol rather than to infer population-level significance over 15 independent real-world scenes.

On ScanNet, HCSG-SLAM is evaluated on five distinct real-world scenes with five paired random seeds per scene. Across the 25 scene–seed pairs, the overall mean mIoU increases from 0.366279 to 0.372166, corresponding to an absolute improvement of 0.005887 and a relative gain of 1.607%, with positive gains in 22 out of the 25 paired runs. The largest scene-level improvement is observed on scene0000_00, where the mean mIoU increases from 0.3149 to 0.3339. Positive mean gains are also observed on scene0059_00, scene0106_00, scene0181_00, and scene0207_00, although the difference on scene0181_00 is marginal.

To avoid treating repeated random seeds as independent scene-level samples, the five paired runs are first averaged within each ScanNet scene and statistical analysis is performed over the resulting five scene-level paired differences. All five scene-level mean differences are positive, with an average improvement of 0.005887. The corresponding 95% confidence interval is [−0.003522, 0.015297]. The paired *t*-test yields *t*(4) = 1.737, *p* = 0.157, while the Wilcoxon signed-rank test yields *W* = 0, *p* = 0.0625, with Cohen’s *d_z_* = 0.777. Therefore, the expanded evaluation shows a consistently positive direction across the five tested scenes, although the scene-level difference does not reach the conventional statistical significance threshold with only five independent scenes. This result supports a positive trend under the current evaluation protocol while indicating that broader scene-level validation remains desirable, see Table 2.

The per-class results show that the semantic improvement is not uniform across categories. The largest positive changes were observed for curtain (+0.0143), wall (+0.0081), desk (+0.0080), and chair (+0.0076), while floor, cabinet, table, window, and sink exhibited smaller positive changes. Door and bathtub remained unchanged, whereas picture (−0.0002) and sofa (−0.0023) showed slight decreases. Therefore, the overall mIoU gain is mainly concentrated in several structural and furniture categories rather than arising from uniform improvement across all semantic classes.

To determine whether the semantic improvements are obtained without noticeably degrading the original SLAM tracking and reconstruction performance, Table 3 reports the corresponding ATE, PSNR, MS-SSIM, and LPIPS results. The Replica results are averaged over the three evaluation scenes, whereas the ScanNet results are averaged over the five evaluation scenes used in the expanded main evaluation.

As shown in Table 3, HCSG-SLAM maintains tracking and reconstruction performance close to that of the Baseline on both datasets. On Replica, the ATE RMSE decreases slightly from 0.1053 cm to 0.1007 cm, while PSNR and MS-SSIM increase from 40.8590 dB and 0.99484 to 41.0253 dB and 0.99619, respectively; LPIPS also decreases from 0.02579 to 0.02511. On ScanNet, the ATE RMSE remains essentially unchanged, decreasing slightly from 1.4522 cm to 1.4519 cm. PSNR changes only from 26.8190 dB to 26.8177 dB, and MS-SSIM from 0.94204 to 0.94196, while LPIPS decreases from 0.11599 to 0.11020. Overall, the tracking and reconstruction metrics remain comparable to those of the Baseline.

Taken together, these results indicate that the semantic gains of HCSG-SLAM are achieved while largely preserving the original SLAM tracking and reconstruction performance. This observation is consistent with the design of HCSG-SLAM, which modifies only the semantic supervision and historical semantic-fusion process while leaving the original RGB/depth tracking and reconstruction objectives unchanged. Therefore, the improvements reported in Table 1 primarily reflect enhanced semantic consistency rather than changes to the underlying geometric tracking or RGB reconstruction pipeline.

To further determine whether the observed semantic improvement can be attributed to the proposed historical representation rather than to generic temporal smoothing, we compared HCSG-SLAM with several alternative temporal semantic-fusion strategies under an identical controlled protocol. All methods used the same three ScanNet scenes, the first 100 frames, seed 101, the same cached semantic predictions, and the same SGS-SLAM tracking and mapping parameters. Therefore, the differences in mIoU mainly reflect the effect of the temporal semantic-fusion strategy rather than differences in semantic input or SLAM configuration.

As shown in Table 4, all temporal-fusion strategies improved the semantic mapping performance relative to the frame-wise Baseline on most of the evaluated scenes. HCSG-SLAM achieved the highest mIoU on scene0059_00, increasing the score from 0.506723 for the Baseline to 0.511290. On scene0000_00, Hist-Maj. obtained the highest mIoU of 0.335898, while HCSG-SLAM achieved a closely comparable value of 0.334487. On scene0106_00, FD-EMA yielded the highest mIoU of 0.373306, followed by Hist-Maj. at 0.371878 and HCSG-SLAM at 0.371701.

Averaging the results over the three ScanNet scenes, the Baseline, SW-Maj., CW-Fusion, FD-EMA, Hist-Maj., and HCSG-SLAM achieved mean mIoU values of 0.396966, 0.399991, 0.402725, 0.404060, 0.405924, and 0.405826, respectively. Hist-Maj. achieved a slightly higher mean mIoU than HCSG-SLAM (0.405924 versus 0.405826), with a difference of only 0.000098. Therefore, this comparison does not demonstrate a semantic accuracy advantage for HCSG-SLAM over Hist-Maj. The reported results are limited to three ScanNet scenes (scene0000_00, scene0059_00, and scene0106_00), the first 100 frames of each scene, and a single random seed (101), using identical cached semantic predictions and SGS-SLAM settings.

In terms of historical-state representation, HCSG-SLAM maintained three scalar variables per Gaussian, whereas Hist-Maj. maintained class-wise accumulated statistics. This distinction describes the structure and dimensionality of the stored temporal states, rather than a measured runtime or total-memory advantage. Table 4 reports semantic accuracy only; the memory and runtime costs of Hist-Maj., Sliding-Window Majority, and Confidence-Weighted Fusion were not evaluated under this matched ScanNet protocol. Consequently, the results establish comparable semantic accuracy under the stated evaluation setting but do not establish a measured efficiency advantage over these alternative temporal-fusion baselines.

These results indicate that the main advantage of HCSG-SLAM lies not in universally outperforming every higher-capacity temporal aggregation strategy, but in achieving competitive semantic accuracy with a substantially more compact historical representation and a strictly causal update mechanism.

To evaluate the sensitivity of HCSG-SLAM to the two hyperparameters controlling history-guided correction, we varied the fusion coefficient *λ* and the warm-up length *n_warm_* under the same controlled ScanNet protocol used above. All experiments used the same three scenes, the first 100 frames, seed 101, and identical cached semantic predictions. When one parameter was varied, the other was fixed at its default value. In the main experiments, we used *λ* = 0.70 and *n_warm_* = 4 consistently across all datasets, scenes, and random seeds, without dataset-specific or scene-specific tuning.

As shown in Table 5, HCSG-SLAM exhibited relatively stable performance over a broad range of fusion coefficients. A very small fusion weight provides insufficient historical influence, whereas moderate-to-high settings produce consistently similar semantic accuracy. We therefore retained *λ* = 0.70 as the default because it provides a history-dominant yet still soft correction, avoiding complete replacement of the current semantic supervision. The results were even less sensitive to n_warm_. Across all tested warm-up settings, the differences in mIoU remained very small, indicating that the method is not strongly dependent on the exact warm-up length. We therefore used n_warm_ = 4 as a short and fixed initialization period that allows several historical observations to be accumulated before history-guided correction is activated, rather than treating it as a parameter requiring scene-specific optimization.

Overall, the sensitivity study indicates that the observed improvement of HCSG-SLAM is not driven by careful tuning of *λ* or *n_warm_*, and the fixed parameter configuration used in the main experiments provides stable performance across the evaluated scenes.

### 4.4. Component-Level Ablation Study

To further examine the contribution of individual components in HCSG-SLAM, we conducted a component-level ablation study under the same controlled ScanNet protocol used in the preceding temporal-fusion and sensitivity experiments. All variants used the same three scenes, the first 100 frames, seed 101, identical cached semantic predictions, and the same SGS-SLAM tracking and mapping settings. Unless the ablated component itself was the target, the default correction parameters were fixed at *λ* = 0.70 and n_warm_ = 4. For the w/o Warm-up variant, n_warm_ was set to 0, while all remaining settings were unchanged. Each variant therefore modifies only the indicated component of the full model. The Candidate-Only variant serves as a last-observation reference: after the current-frame correction target has been constructed, each valid Gaussian stores the latest valid semantic observation as its candidate without accumulating persistent voting support across frames.

As shown in Table 6, removing the voting accumulation mechanism caused the most pronounced performance degradation. The mean mIoU decreased from 0.405826 for the full HCSG-SLAM to 0.397848 for the Candidate-Only variant. This result indicates that persistent cross-frame accumulation of semantic support, rather than merely retaining the current candidate label, is the primary contributor to the semantic improvement achieved by the proposed historical state.

In contrast, the w/o Warm-up variant achieved a mean mIoU of 0.405820, which is virtually identical to the full configuration and is consistent with the weak sensitivity to n_warm_ observed in Table 5. Similarly, removing the conflict gate results in a mean mIoU of 0.405822, differing from the full model by only 0.000004. This indicates that the conflict gate is not a major source of average accuracy improvement, but instead serves to restrict history-guided correction to locations where the current semantic prediction disagrees with the historical consensus. The w/o Observation Count variant obtained a slightly higher mean mIoU of 0.405918; however, the difference remained marginal. This is consistent with the implemented HCSG-SLAM formulation, in which the observation count serves as a bookkeeping and diagnostic state for valid historical semantic observations, rather than an independent gating criterion or a factor in the final semantic correction weight.

Hard replacement yielded a slightly lower mean mIoU of 0.405637, indicating that completely overwriting the current semantic supervision with the historical target does not provide an advantage over the proposed soft correction. The Non-Causal Update variant remained essentially unchanged at 0.405834. Therefore, the strictly causal target-before-current-update strategy was retained primarily to prevent current-frame self-reinforcement and to maintain a well-defined online update process, rather than because it produces a large standalone accuracy gain. Overall, the ablation results show that the main semantic improvement is primarily driven by persistent Gaussian-level candidate-and-vote accumulation, while the remaining components mainly regulate the initialization, selectivity, state bookkeeping, and causal structure of history-guided correction.

### 4.5. Efficiency and Robustness Analysis

In this work, noisy semantic predictions primarily refer to imperfect frame-wise outputs produced by the semantic segmentation network, while controlled semantic corruption is used only as an additional stress test. To further evaluate the trade-off among semantic accuracy, temporal-state compactness, runtime overhead, and whole-process GPU memory, HCSG-SLAM was compared with the full-distribution FD-EMA reference. Table 7 summarizes the semantic accuracy, additional temporal semantic-state memory, runtime, and peak GPU-memory measurements of the three configurations.

As shown in Table 7, HCSG-SLAM achieved a mean mIoU improvement of 0.006180 with positive gains in all 15 paired Replica runs, whereas FD-EMA achieved a mean improvement of 0.005170 with positive gains in 13 out of the 15 pairs. Under the current paired Replica protocol, the semantic-accuracy difference between HCSG-SLAM and FD-EMA was not statistically significant (paired *t*-test, *p* = 0.162; Wilcoxon signed-rank test, *p* = 0.252), indicating comparable semantic performance. In terms of temporal semantic-state storage, HCSG-SLAM requires only three float32 scalars, corresponding to 12 B per Gaussian, whereas FD-EMA maintains a complete 101-class float16 distribution requiring approximately 202 B per Gaussian. The corresponding temporal-state memory is 19.54 MiB for HCSG-SLAM and 328.93 MiB for FD-EMA, meaning that FD-EMA requires approximately 16.83 times as much temporal semantic-state memory.

Runtime statistics aggregated over the 15 formal Replica runs further show that the average tracking times were 5.299, 5.297, and 5.314 s/frame for the Baseline, HCSG-SLAM, and FD-EMA, respectively, while the corresponding mapping times were 9.137, 9.324, and 9.360 s/frame. Relative to the Baseline, the additional mapping overhead was therefore approximately 2.05% for HCSG-SLAM and 2.44% for FD-EMA. The tracking time of HCSG-SLAM remained essentially unchanged relative to the Baseline, while FD-EMA introduces only a small increase.

Because the main SLAM experiments used cached semantic predictions, the tracking and mapping times reported above do not include DeepLabV3 inference. We therefore separately benchmarked semantic inference over 300 Replica frames from room0, office0, and room1. The measured DeepLabV3-ResNet50 inference latency was 21.734 ± 0.675 ms/frame, with a median of 21.846 ms/frame and a 95th-percentile latency of 22.633 ms/frame. When this measured inference time is serially combined with the tracking and mapping times, the resulting semantic-inference-inclusive processing times were 14.4577 s/frame for the Baseline, 14.6427 s/frame for HCSG-SLAM, and 14.6957 s/frame for FD-EMA. Under this serial composition, HCSG-SLAM introduced an overall increase of approximately 1.280% relative to the Baseline, compared with 1.646% for FD-EMA. Since semantic inference and SLAM were benchmarked separately, these values are reported as semantic-inference-inclusive serial latency rather than directly measured integrated end-to-end runtime.

To complement the semantic-state analysis, total process-level peak GPU memory was additionally measured under an identical representative profiling protocol. The measured peak memory was 4146 MiB for the Baseline, 4338 MiB for HCSG-SLAM, and 4266 MiB for FD-EMA. Thus, although HCSG-SLAM uses a substantially smaller temporal semantic-state representation than FD-EMA, this reduction does not directly translate into the lowest whole-process peak GPU memory in the current implementation. The peak memory of HCSG-SLAM was approximately 4.63% higher than that of the Baseline and approximately 1.69% higher than that of FD-EMA. These measurements characterize a different aspect of efficiency from the temporal-state memory reported above: temporal-state memory isolates the storage cost introduced by the semantic-history representation, whereas peak GPU memory reflects the complete runtime footprint of the SLAM process. Accordingly, the term lightweight in this work specifically refers to the compact, fixed-size temporal semantic-state representation and the modest additional fusion overhead, rather than to the minimum total GPU memory consumption.

Overall, Table 7 shows that HCSG-SLAM provides semantic accuracy comparable to the higher-capacity FD-EMA reference while requiring substantially less temporal semantic-state storage and only a modest increase in mapping time. The whole-process GPU-memory results further clarify that the principal efficiency advantage of HCSG-SLAM lies in the compact representation of temporal semantic history rather than in reducing the total memory footprint of the complete SLAM system. It should be noted that the memory and runtime comparison in Table 7 concerns the Baseline, HCSG-SLAM, and FD-EMA under the separate Replica evaluation and profiling protocols. It does not constitute a matched efficiency comparison with all temporal-fusion baselines listed in Table 4.

In addition to state efficiency, we evaluated the robustness of HCSG-SLAM to controlled perturbations in the network semantic input. For the controlled transient-corruption tests, a deterministic frame-wise corruption mask was generated at target ratios of 10%, 20%, and 30% using the fixed experimental seed together with the frame index. At selected pixels, the semantic label was replaced by the label obtained from a spatially shifted version of the same frame-wise semantic prediction, with the displacement varying deterministically across frames. The identical corrupted prediction cache was used by both the Baseline and HCSG-SLAM.

Positive mIoU gains were observed under all nine controlled corruption conditions across the three ScanNet scenes. On scene0000_00, the relative gain increased from 7.792% at 10% corruption to 10.432% and 15.047% at 20% and 30% corruption, respectively. On scene0059_00, the relative gain increased from 1.558% to 2.286% and 3.607% as the corruption level increased. For scene0106_00, the gains were smaller but remained consistently positive at 0.539%, 0.785%, and 0.815%.

These results should not be interpreted as indicating that stronger semantic corruption inherently improves HCSG-SLAM. Rather, under the controlled corruption protocol, the Baseline generally degraded faster than HCSG-SLAM, resulting in an increased relative advantage at higher corruption levels. The results therefore indicate that the proposed historical correction improves robustness to the evaluated semantic perturbations, although the magnitude of the improvement varies across scenes.

To further evaluate robustness beyond the controlled transient corruption in Table 8, we additionally considered two more challenging semantic error patterns: persistent class confusion and structured boundary shift. For both tests, corrupted semantic predictions were generated offline from the same fixed DeepLabV3 outputs before SLAM execution, and the identical corrupted inputs were shared by the Baseline and HCSG-SLAM. In the persistent-confusion setting, semantic class 5 was consistently remapped to class 7 over the evaluated 100-frame sequence. In the boundary-shift setting, frame 0 was retained unchanged, while frames 1–99 were generated using a fixed 3-pixel horizontal displacement to the right. Because labels inside homogeneous semantic regions often remain unchanged after the displacement, the actual label changes are concentrated near semantic boundaries; across the three evaluated scenes, the mean fraction of pixels whose semantic labels change was approximately 1.74%. All experiments used the same three ScanNet scenes and seed 101.

As shown in Table 9, HCSG-SLAM also remained effective under persistent and spatially structured semantic errors. Under persistent class confusion, the three-scene mean mIoU increased from 0.315644 to 0.322406, corresponding to an absolute gain of 0.006762. Positive mean differences were observed on all three scenes, although the change on scene0106_00 was marginal. Under the structured boundary-shift perturbation, HCSG-SLAM improved the mean mIoU from 0.397653 to 0.407799, with an absolute gain of 0.010146, and positive gains were observed on all three scenes. These results indicate that the robustness of HCSG-SLAM is not limited to the transient corruption setting in Table 8, and that the proposed historical correction can also maintain positive or comparable performance under persistent class confusion and spatially structured boundary errors.

Despite the overall positive trend, HCSG-SLAM did not improve every individual scene–seed run. A representative failure case occurred on ScanNet scene0181_00 with seed 101, where the mean mIoU decreased from 0.408138 for the Baseline to 0.406272 for HCSG-SLAM, corresponding to ∆*mIoU* = −0.001866. Among the 21 evaluated frames in this run, 2 frames improved, 17 frames decreased, and 2 remained unchanged. The largest frame-level decrease occurred at frame 44, where the mIoU changed from 0.335057 to 0.327385 ∆ = −0.007671. This case indicates that history-guided correction does not guarantee improvement for every observation sequence and may occasionally introduce unfavorable semantic modifications. Therefore, HCSG-SLAM should be interpreted as improving semantic consistency on average under the evaluated conditions rather than monotonically improving every scene, seed, or frame.

To further examine the mechanism underlying these robustness gains, we analyzed several diagnostic quantities recorded during semantic correction.

The semantic conflict ratio measures the proportion of valid observations for which the current network prediction disagrees with the historical consensus, whereas the corrected ratio measures the proportion of observations for which history-guided correction is actually activated. In addition, historical confidence is used as a diagnostic quantity to characterize the stability of the accumulated historical semantic consensus. These quantities are used only for behavior analysis and are not employed as hard gating conditions in the final HCSG-SLAM method.

As the controlled semantic corruption becomes stronger, both the semantic conflict ratio and the corrected ratio generally increase, while historical confidence tends to decrease. For example, on scene0000_00, the corrected ratio increased from approximately 0.205 at 10% corruption to 0.279 at 20% corruption and 0.357 at 30% corruption. This trend indicates that HCSG-SLAM invokes historical information more frequently as transient semantic errors become more prevalent. At the same time, the decrease in historical confidence suggests that stronger input corruption also weakens the reliability of the accumulated historical consensus.

Overall, these diagnostic responses are consistent with the observed robustness results: the correction mechanism becomes more active as semantic conflicts increase, while the quality of the historical evidence itself gradually deteriorates under stronger perturbations. This behavior helps explain both the positive mIoU gains under controlled corruption and the variation in gain magnitude across different scenes.

**Figure 3:**
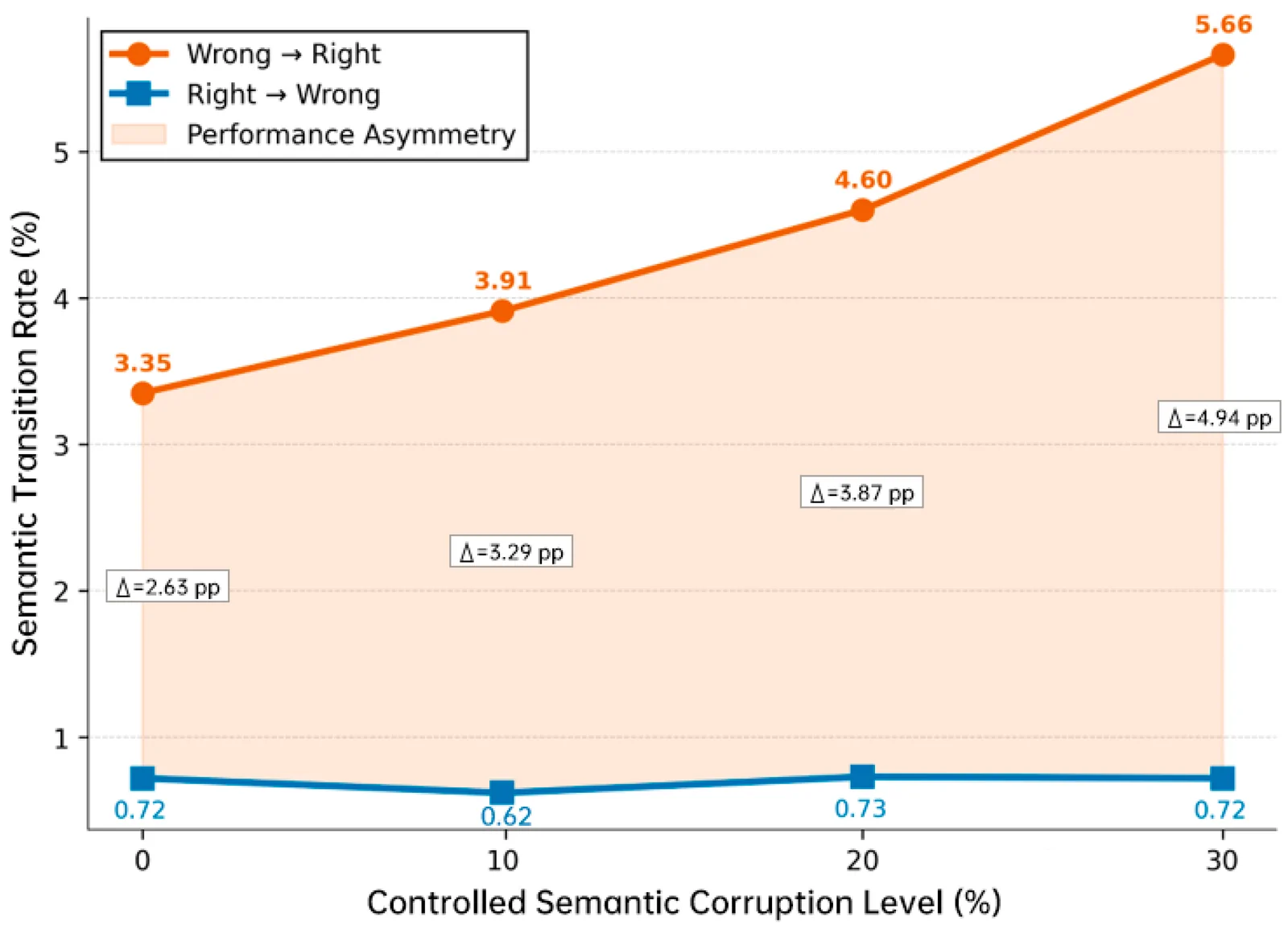
Wrong-to-Right and Right-to-Wrong semantic transition rates on scene0000_00 under different controlled corruption levels.

To further quantify whether the semantic modifications introduced by HCSG-SLAM correspond to beneficial corrections or newly introduced errors, we analyzed the pixel-level transition directions between the Baseline and HCSG-SLAM. Figure 3 reports the Wrong-to-Right (W→R) and Right-to-Wrong (R→W) transition rates on scene0000_00 under different controlled semantic corruption levels.

Under the clean condition, the W→R and R→W transition rates were 3.345% and 0.718%, respectively, corresponding to a net correction difference of +2.627 percentage points. As the corruption level increases, the W→R rate rises consistently, whereas the R→W rate remains comparatively low. At 30% corruption, W→R reached 5.658%, while R→W remained approximately 0.719%, increasing the net correction difference to approximately +4.94 percentage points.

These results indicate that the robustness gains under controlled semantic corruption are primarily associated with correcting previously incorrect predictions rather than with a comparable increase in newly introduced errors. Together with the diagnostic quantities discussed above, the transition analysis shows that stronger semantic perturbations trigger more frequent historical correction, while the proportion of adverse corrections remains relatively low. This provides a complementary explanation for the positive mIoU gains observed under the evaluated corruption protocol.

### 4.6. Qualitative Results and 3D Gaussian Maps

**Figure 4:**
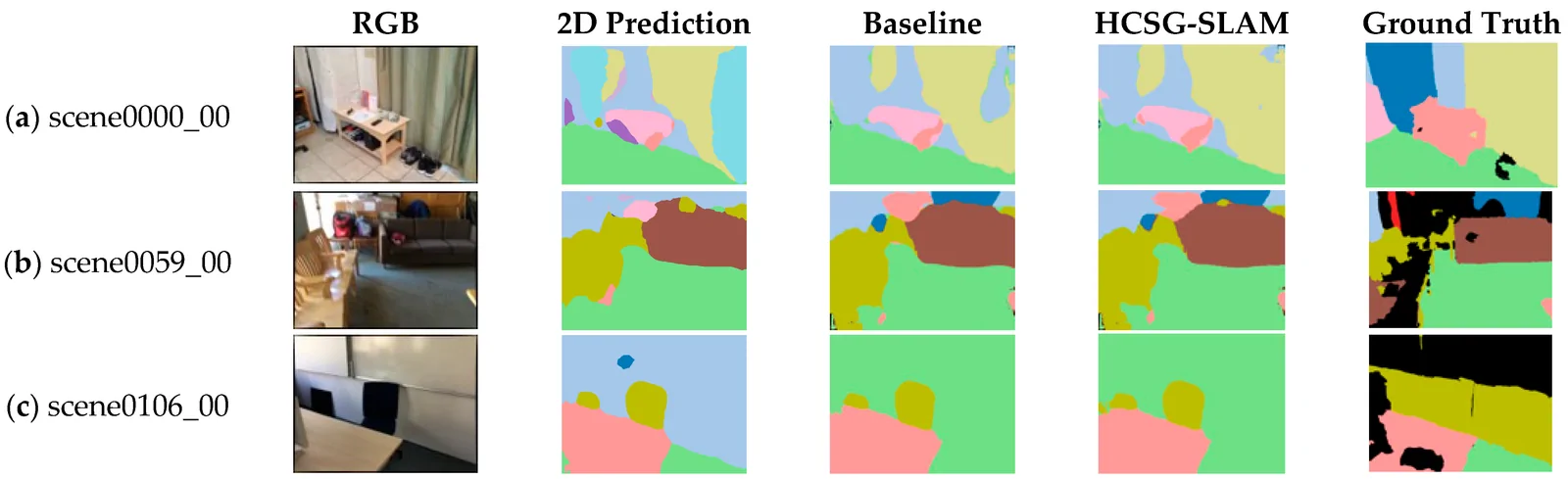
Qualitative semantic mapping comparisons on ScanNet: (**a**) scene0000_00; (**b**) scene0059_00; (**c**) scene0106_00. Columns from left to right show the RGB image, 2D semantic prediction, Baseline result, HCSG-SLAM result, and ground truth.

**Figure 5:**
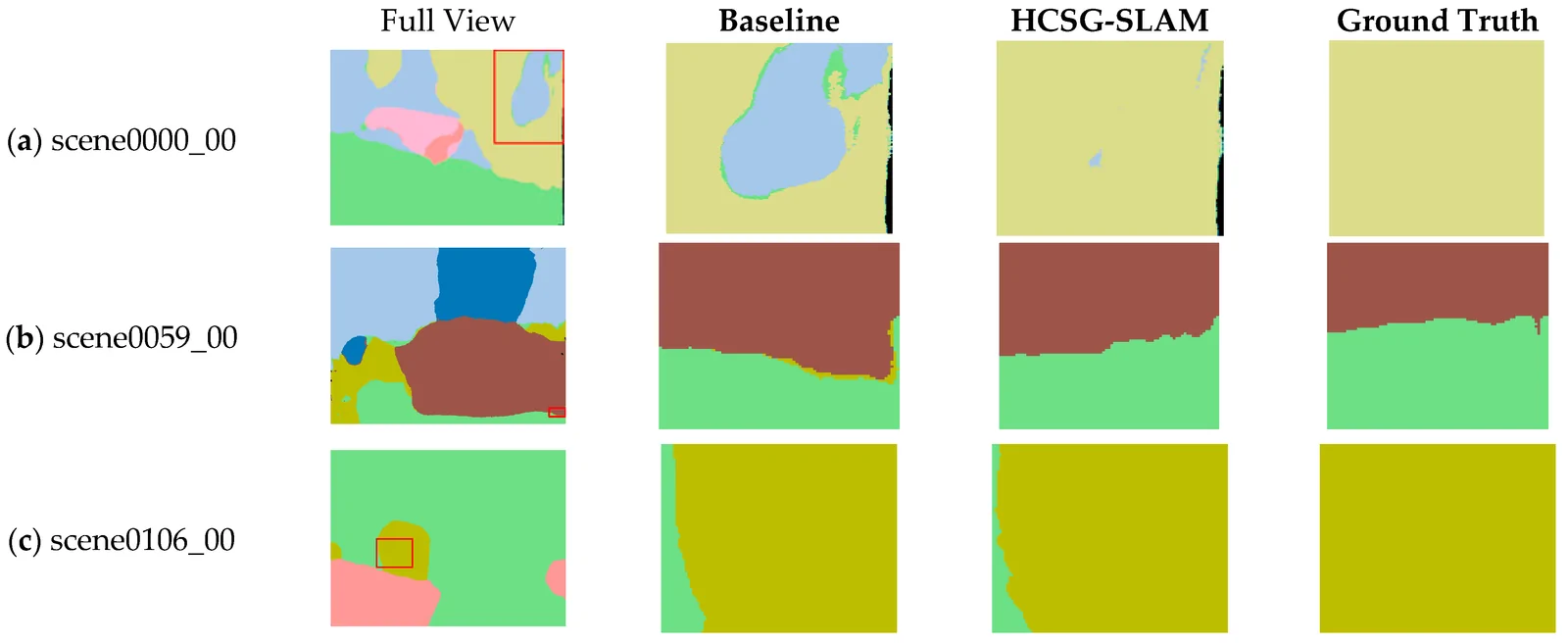
Representative local semantic correction examples of HCSG-SLAM on ScanNet: (**a**) scene0000_00; (**b**) scene0059_00; (**c**) scene0106_00. Columns from left to right show the full-view semantic map with the selected region, Baseline result, HCSG-SLAM result, and ground truth.

**Figure 6:**
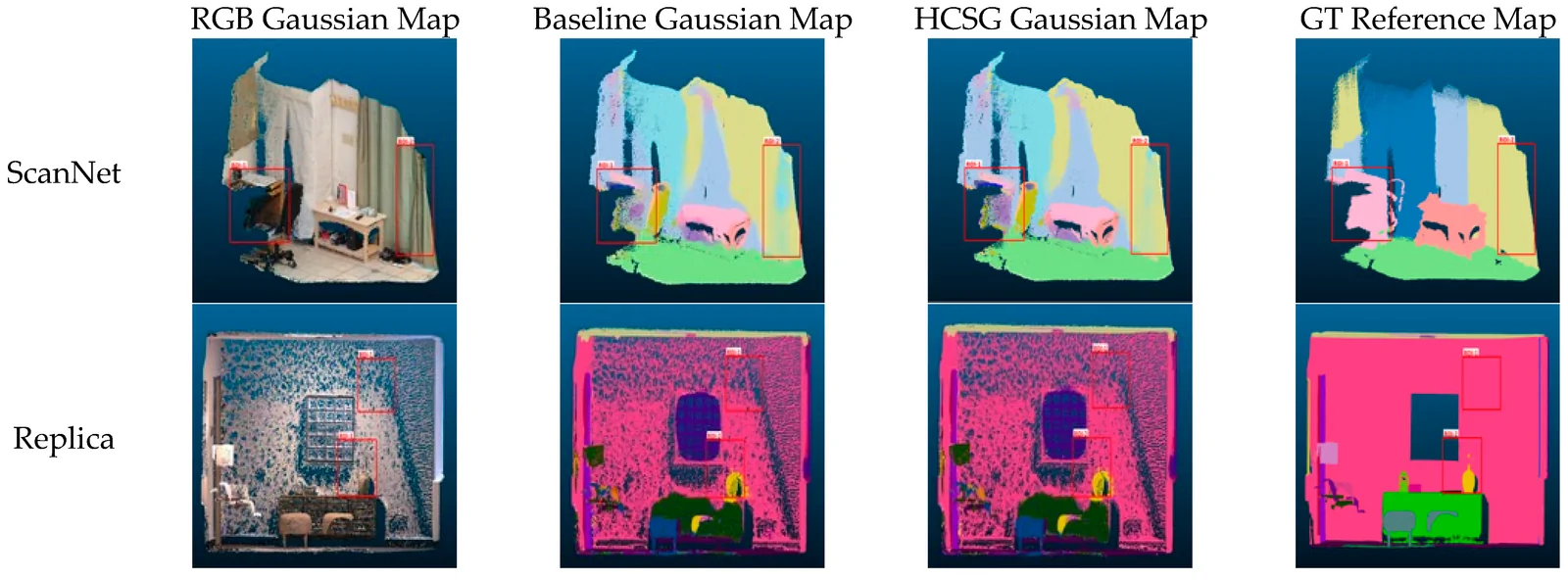
Qualitative comparison of reconstructed 3D Gaussian maps on ScanNet and Replica. The top row shows the ScanNet scene and the bottom row shows the Replica scene. From left to right, the columns show the RGB Gaussian map, the Baseline semantic Gaussian map, the HCSG-SLAM semantic Gaussian map, and the ground-truth semantic reference. ROI-1 and ROI-2 highlight representative regions where the Baseline and HCSG-SLAM exhibit different semantic reconstructions relative to the ground truth.

Quantitative metrics characterize the overall semantic performance but do not directly reveal where semantic corrections occur or whether the final output is represented in the 3D Gaussian map. We therefore provide qualitative comparisons at both the image and map levels. Figure 4, Figure 5 and Figure 6 show the overall semantic mapping results, representative local correction regions, and reconstructed RGB/semantic 3D Gaussian maps, respectively.

The qualitative results are consistent with the quantitative trends in Table 1. HCSG-SLAM preserves the dominant semantic structure produced by the Baseline while correcting a subset of inconsistent regions. The magnitude and spatial extent of the corrections vary across scenes, consistent with the variation in mIoU improvement observed across the evaluated ScanNet scenes in Section 4.3.

To examine the corrections more closely, Figure 5 enlarges representative regions in which the Baseline and HCSG-SLAM produce different semantic outputs.

These examples illustrate that the history-guided correction is spatially selective rather than uniformly applied to the entire image. In the highlighted regions, HCSG-SLAM suppresses transient semantic inconsistencies while preserving surrounding regions that do not satisfy the correction conditions.

To verify that the semantic improvements are reflected in the final 3D Gaussian representation rather than being limited to image-space post-processing, Figure 6 directly compares the reconstructed RGB Gaussian map, the Baseline semantic Gaussian map, the HCSG-SLAM semantic Gaussian map, and the corresponding ground-truth semantic reference on representative ScanNet and Replica scenes. Two representative regions of interest (ROIs) are highlighted in each scene to facilitate local comparison of the reconstructed 3D semantic structures.

As shown in Figure 6, the semantic information produced by HCSG-SLAM is stored directly in the reconstructed 3D Gaussian representation rather than being applied only as an image-space post-processing operation. Compared with the Baseline semantic Gaussian maps, HCSG-SLAM preserves the overall scene structure while modifying a limited number of locally inconsistent semantic regions. The highlighted ROIs provide a direct comparison with the corresponding ground-truth semantic reference. In both the ScanNet and Replica examples, the HCSG-SLAM reconstruction reduces locally fragmented or inconsistent semantic assignments observed in the Baseline and produces a semantic structure that is visually more consistent with the reference map in the selected regions. Importantly, these corrections remain spatially localized rather than altering the dominant semantic layout of the reconstructed scene. Together with the quantitative mIoU results and the pixel-level transition analysis, the 3D Gaussian-map comparison provides complementary evidence that the proposed history-consistent correction can improve semantic consistency at the map level while preserving the underlying RGB/depth Gaussian reconstruction pipeline.

## 5. Conclusions

This work addresses the propagation of transient two-dimensional semantic segmentation errors into three-dimensional semantic maps in online Gaussian SLAM. We proposed HCSG-SLAM, a lightweight history-consistent semantic fusion method that introduces a compact Gaussian-level historical semantic state into SGS-SLAM without modifying its main RGB/depth tracking and reconstruction pipeline. Each Gaussian maintains only a candidate semantic label, a voting state, and a historical observation count, while a strictly causal target-before-current-update strategy ensures that current-frame semantic correction relies only on previously accumulated historical information.

Experiments on Replica and ScanNet show that HCSG-SLAM generally improves semantic mapping performance when segmentation-network predictions are used as semantic input. On Replica, the mean mIoU increased from 0.300830 to 0.307010, with positive gains in all 15 paired runs. On ScanNet, the evaluation was extended to five scenes, where the overall mean mIoU increased from 0.366279 to 0.372166, with positive gains in 22 out of 25 paired scene–seed runs. All five scene-level mean differences were positive, although the scene-level statistical test did not reach the conventional significance threshold with only five scenes. Per-class analysis further showed that the improvement was concentrated in several structural and furniture categories rather than being uniform across all classes. Positive or comparable performance was also observed under transient, persistent, and spatially structured semantic perturbations, although an individual failure case confirmed that improvement is not guaranteed for every scene–seed sequence.

The component-level ablation study indicates that persistent Gaussian-level candidate-and-vote accumulation is the main contributor to the observed mIoU improvement. In contrast, warm-up, conflict gating, observation counting, and causal ordering show negligible independent effects on mean mIoU under the controlled three-scene protocol. These components primarily support initialization, selective correction, diagnostic bookkeeping, and causal consistency, respectively, rather than providing independently demonstrated accuracy gains.

Compared with FD-EMA, HCSG-SLAM achieves comparable semantic accuracy while requiring only 12 B of additional temporal state per Gaussian, whereas FD-EMA requires approximately 202 B. The corresponding temporal semantic-state memory is 19.54 MiB and 328.93 MiB, respectively. HCSG-SLAM introduces approximately 2.05% additional mapping time relative to the Baseline while leaving tracking essentially unchanged. The total process-level peak GPU memory is not the lowest for HCSG-SLAM; therefore, the lightweight characteristic of the proposed method specifically refers to its compact fixed-size temporal semantic representation and modest fusion overhead rather than to minimum whole-process GPU-memory consumption. The comparison with alternative temporal-fusion baselines beyond FD-EMA is limited to semantic accuracy on three ScanNet scenes using 100 frames per scene and a single random seed; their corresponding runtime and memory costs remain to be systematically evaluated under matched conditions. Future work will investigate more adaptive historical-reliability estimation and broader validation in long-term and dynamic environments.

## Figures and Tables

**Table 1:** Five-seed quantitative results on Replica and ScanNet in terms of mIoU.

Dataset	Scene	Baseline	HCSG-SLAM	ΔmIoU	Relative	Wins
Replica	room0	0.2634 ± 0.0021	0.2735 ± 0.0003	+0.0101	+3.83%	5/5
	office0	0.2535 ± 0.0007	0.2576 ± 0.0006	+0.0041	+1.63%	5/5
	room1	0.3856 ± 0.0006	0.3899 ± 0.0008	+0.0043	+1.12%	5/5
	Overall	0.300830	0.307010	+0.006180	+2.054%	15/15
ScanNet	scene0000_00	0.3149 ± 0.0047	0.3339 ± 0.0011	+0.0189	+6.02%	5/5
	scene0059_00	0.5040 ± 0.0032	0.5097 ± 0.0019	+0.0057	+1.12%	5/5
	scene0106_00	0.3707 ± 0.0018	0.3726 ± 0.0011	+0.0020	+0.53%	4/5
	scene0181_00	0.4065 ± 0.0010	0.4065 ± 0.0005	+0.00003	+0.01%	3/5
	scene0207_00	0.2353 ± 0.0014	0.2381 ± 0.0009	+0.0028	+1.20%	5/5
	Overall	0.366279	0.372166	+0.005887	+1.607%	22/25

**Table 2:** Per-class IoU comparison on the five ScanNet scenes using the five-seed protocol.

Class	Scenes	Baseline IoU	HCSG-SLAM IoU	ΔIoU	Positive Scenes
curtain	3	0.2289	0.2433	+0.0143	2/3
wall	5	0.4359	0.4440	+0.0081	5/5
desk	2	0.0092	0.0171	+0.0080	1/2
chair	3	0.2660	0.2736	+0.0076	3/3
floor	3	0.6664	0.6698	+0.0033	1/3
cabinet	4	0.2026	0.2054	+0.0028	3/4
table	3	0.4429	0.4453	+0.0024	2/3
window	2	0.0083	0.0087	+0.0004	2/2
sink	2	0.0079	0.0080	+0.0001	1/2
door	2	0.0000	0.0000	0.0000	0/2
bathtub	1	0.0000	0.0000	0.0000	0/1
picture	1	0.4441	0.4439	−0.0002	0/1
sofa	1	0.7865	0.7842	−0.0023	0/1

**Table 3:** Tracking and reconstruction metrics on Replica and ScanNet.

Dataset	Method	ATE RMSE (cm)	PSNR (dB)	MS-SSIM	LPIPS
Replica	Baseline	0.1053	40.8590	0.99484	0.02579
	HCSG-SLAM	0.1007	41.0253	0.99619	0.02511
ScanNet	Baseline	1.4522	26.8190	0.94204	0.11599
	HCSG-SLAM	1.4519	26.8177	0.94196	0.11020

**Table 4:** Controlled semantic accuracy comparison of temporal-fusion strategies on three ScanNet scenes using the first 100 frames per scene and a single random seed (101).

Scene	Baseline	SW-Maj. (W = 5)	CW-Fusion	FD-EMA	Hist-Maj.	HCSG-SLAM
scene0000_00	0.315105	0.321234	0.328158	0.330058	0.335898	0.334487
scene0059_00	0.506723	0.509472	0.510023	0.508816	0.509997	0.511290
scene0106_00	0.369069	0.369266	0.369994	0.373306	0.371878	0.371701

**Table 5:** Sensitivity of HCSG-SLAM to the fusion coefficient *λ* and warm-up length *n*_warm_ on three ScanNet scenes using seed 101.

Parameter	Setting	scene0000_00	scene0059_00	scene0106_00	Mean
*λ*	0.3	0.323178	0.508971	0.370087	0.400745
	0.5	0.331448	0.510771	0.370801	0.404340
	0.7	0.334487	0.511290	0.371701	0.405826
	0.9	0.335233	0.509128	0.371884	0.405415
	1.0	0.336277	0.508712	0.371923	0.405637
*n_warm_*	0	0.334658	0.511362	0.371438	0.405820
	2	0.334522	0.511544	0.371359	0.405808
	4	0.334487	0.511290	0.371701	0.405826
	8	0.334936	0.511314	0.371241	0.405831

**Table 6:** Component-level ablation of HCSG-SLAM on three ScanNet scenes using the first 100 frames and seed 101.

Configuration	scene0000_00	scene0059_00	scene0106_00	Mean
Baseline	0.315105	0.506723	0.369069	0.396966
Candidate Only	0.316983	0.508713	0.367848	0.397848
w/o Warm-up	0.334658	0.511362	0.371438	0.405820
Hard Replacement	0.336277	0.508712	0.371923	0.405637
Non-Causal Update	0.334211	0.512003	0.371288	0.405834
HCSG-SLAM (Full)	0.334487	0.511290	0.371701	0.405826
w/o Conflict Gate	0.334448	0.511649	0.371369	0.405822
w/o Observation Count	0.334735	0.511618	0.371402	0.405918

**Table 7:** Accuracy, temporal semantic-state memory, runtime, and peak GPU-memory comparison.

Method	Mean ΔmIoU	Positive Pairs	Additional Temporal State/Gaussian	Temporal-State Memory	Peak GPU (MiB)	Tracking/Mapping (s/Frame)
Baseline	0	—	—	—	4146	5.299/9.137
HCSG-SLAM	+0.006180	15/15	3 × float32 = 12 B	19.54 MiB	4338	5.297/9.324
FD-EMA	+0.005170	13/15	101 × float16 ≈ 202 B	328.93 MiB	4266	5.314/9.360

**Table 8:** Robustness under controlled semantic corruption on ScanNet (mIoU).

Scene	Corruption	Baseline	HCSG-SLAM	ΔmIoU	Relative
scene0000_00	10%	0.3080	0.3320	+0.0240	+7.792%
	20%	0.2943	0.3250	+0.0307	+10.432%
	30%	0.2738	0.3150	+0.0412	+15.047%
scene0059_00	10%	0.5007	0.5085	+0.0078	+1.558%
	20%	0.4944	0.5057	+0.0113	+2.286%
	30%	0.4769	0.4941	+0.0172	+3.607%
scene0106_00	10%	0.3708	0.3728	+0.0020	+0.539%
	20%	0.3692	0.3721	+0.0029	+0.785%
	30%	0.3679	0.3709	+0.0030	+0.815%

**Table 9:** Robustness under persistent and structured semantic perturbations on ScanNet.

Perturbation	Scene	Baseline	HCSG-SLAM	ΔmIoU	Relative
Persistent 5→7	scene0000_00	0.320709	0.337017	+0.016308	+5.085%
	scene0059_00	0.380533	0.384471	+0.003939	+1.035%
	scene0106_00	0.245690	0.245728	+0.000039	+0.016%
	Mean	0.315644	0.322406	+0.006762	+2.142%
Boundary Shift	scene0000_00	0.314597	0.335847	+0.021250	+6.755%
	scene0059_00	0.510891	0.516094	+0.005204	+1.019%
	scene0106_00	0.367472	0.371457	+0.003985	+1.084%
	Mean	0.397653	0.407799	+0.010146	+2.552%

## Data Availability

The Replica and ScanNet datasets used in this study are publicly available from their respective dataset repositories. The additional experimental data generated during this study are available from the first author upon reasonable request.
